# Persuasive Design in a Digital Mindfulness Intervention: A Randomized Trial of a Skill-Based Achievement System and Automated Peer Encouragement

**DOI:** 10.1007/s41666-025-00216-6

**Published:** 2025-10-29

**Authors:** Abdul Rahman Idrees, Robin Kraft, Ann-Marie Küchler, Leandra Bantleon, Harald Baumeister, Manfred Reichert, Fanny Kählke, David Daniel Ebert, Rüdiger Pryss

**Affiliations:** 1 Institute of Databases and Information Systems, Ulm University, Ulm, Germany; 2 Department of Clinical Psychology and Psychotherapy, Ulm University, Ulm, Germany; 3 Institute of Clinical Epidemiology and Biometry, University of Würzburg, Würzburg, Germany; 4 Institute of Medical Data Science, University Hospital Würzburg, Würzburg, Germany; 5 Faculty of Applied Health Sciences, Deggendorf Institute of Technology, Deggendorf, Germany; 6 Professorship of Psychology and Digital Mental Health Care, Department of Sports and Health Sciences, Technical University of Munich (TUM), Munich, Germany

**Keywords:** eHealth, Persuasive design, Adherence, Gamification, Usability, Internet-interventions

## Abstract

Adherence to digital health interventions remains a persistent challenge, limiting their effectiveness and motivating the need for scalable strategies that promote sustained engagement. This randomized controlled trial investigated whether user adherence could be enhanced by two specific persuasive design strategies: a gamification approach focused on visualizing skill progression, and a social support approach based on automated interaction between supportive partners. University students were recruited and assigned to a control, gamification, or social support group. The control group received the standard intervention without persuasive strategies, whereas the gamification group utilized skill-based progression systems and infographics. In the social support group, participants were paired into teams. Participants were encouraged to motivate teammates to complete daily diary entries, with mutual completion triggering a congratulatory email. Average module completions were: control ($\boldsymbol{M = 2.52}$, *SD* = 1.92), gamification ($\boldsymbol{M = 2.69}$, *SD* = 1.89), and social support ($\boldsymbol{M = 2.76}$, *SD* = 2.03). Neither the gamification components nor the automated peer support system improved adherence compared to the control. Although the peer support strategy in the social support group recorded more diary entries, this did not translate to improved module adherence. User experience ratings were comparable in all groups, indicating that the strategies did not affect usability. The smaller-than-planned sample size and higher attrition rates warrant cautious interpretation of these findings. These results contribute to healthcare informatics by demonstrating the limitations of generic persuasive strategies and highlighting the need for more adaptive, context-aware engagement mechanisms in digital interventions.

## Introduction

Internet- and mobile-based interventions (IMIs) have emerged as promising tools for delivering healthcare interventions, psychological support, and behavior change programs to a wide range of populations [1, 2]. These interventions offer several advantages, including accessibility, scalability, and cost-effectiveness, making them particularly suitable for reaching individuals who may face barriers to accessing traditional forms of support [3, 4]. However, despite the potential benefits of IMIs, their effectiveness can be hindered by challenges such as low adherence and engagement, high attrition rates, and limited behavior change outcomes [5–7]. One method of addressing these challenges is the application of persuasive design (PD) principles [8]. PD principles are rooted in the understanding that behavior change can be facilitated through strategic design choices. Fogg’s Behavior Model (FBM) presents three key factors in influencing behavior: motivation, ability, and triggers. Motivation is the desire to perform a behavior, ability is the ease with which it can be performed, and triggers are the cues that initiate them. Based on the FBM, behavior change occurs when motivation, ability, and triggers converge at the same time [9]. Building on FBM, PD aims to enhance motivation and perceived ability through tailored strategies while providing effective triggers, thereby facilitating behavior change [9]. In practice, PD optimization systematically integrates persuasive strategies into the design and delivery of IMIs, with the goal of maximizing their impact on user behavior and outcomes [8]. These strategies draw on principles from psychology, behavioral economics, and human-computer interaction (HCI) to enhance user engagement, motivation, and adherence to intervention protocols [10]. Two persuasive strategies that have come to the forefront in the design of IMIs are gamification and social support. Gamification refers to the use of game-like elements, such as points, badges, and leaderboards, in non-game contexts [11]. It aims to make interactions more engaging by incorporating features commonly found in games, which can include progress bars, levels, challenges, and rewards [11]. Social support involves the integration of features that encourage users to engage with their intervention, often through peer interactions or system-driven prompts that promote accountability and adherence [12]. While there is growing interest in the use of PD in IMIs, there remains a need for greater empirical research to evaluate its effectiveness and identify best practices for implementation. This is particularly pertinent as most RCTs do not explicitly examine adherence or engagement as primary outcomes. Randomized controlled trials (RCTs) are widely regarded as a reliable method for evaluating the efficacy of interventions and can provide evidence of their effects on specific outcomes [13]. Additionally, RCTs are an excellent framework for so-called component studies that use experimental designs to compare standard interventions to interventions with added components. Such designs can also be used to investigate the efficacy of PD features [14]. The results of a three-armed RCT are presented in this paper, which examines an IMI (i.e., StudiCare Mindfulness) aimed at promoting mindfulness among university students. A previous evaluation has shown the effectiveness of StudiCare Mindfulness in improving university students’ mental health. However, intervention adherence was suboptimal, with only 20 to 30 percent of participants completing the core modules [15]. Consequently, this study explores the impact of two specific PD optimization strategies on user adherence and engagement: a gamification strategy utilizing a skill system and progress infographics, and a social support strategy using an automated system of email reminders and congratulatory messages. Furthermore, the study aims to explore the impact of these specific strategies on both the usability of the StudiCare Mindfulness intervention and the eHealth platform that delivers it. It explores how these strategies affect user experience within digital interventions targeting mental well-being. More specifically, the primary aim and additional exploratory objectives of this RCT are as follows:

1. Primary Aim and Hypothesis:
  Research Question: Does PD optimization (i.e., the gamification or social support strategies implemented) influence user adherence to the intervention compared to a standard version of the intervention?
  Hypothesis: Participants in the gamification group (GG) and social support group (SSG) will demonstrate significantly higher adherence to the intervention compared to participants in the Control Group (CG).
2. Exploratory Objectives:
  To examine whether PD optimization (i.e., the gamification or social support strategies implemented) influences user engagement with the intervention compared to a standard version.
  To explore potential differences between gamification and social support in their effects on user adherence and engagement.
  To investigate the association between the number of diaries completed (brief daily journaling tasks) and overall module completion.
  To assess whether PD optimization (i.e., the gamification or social support strategies implemented) influences the usability of the eSano eHealth platform in the context of the StudiCare Mindfulness intervention.

## Methods

### Trial Design

**Fig. 1:**
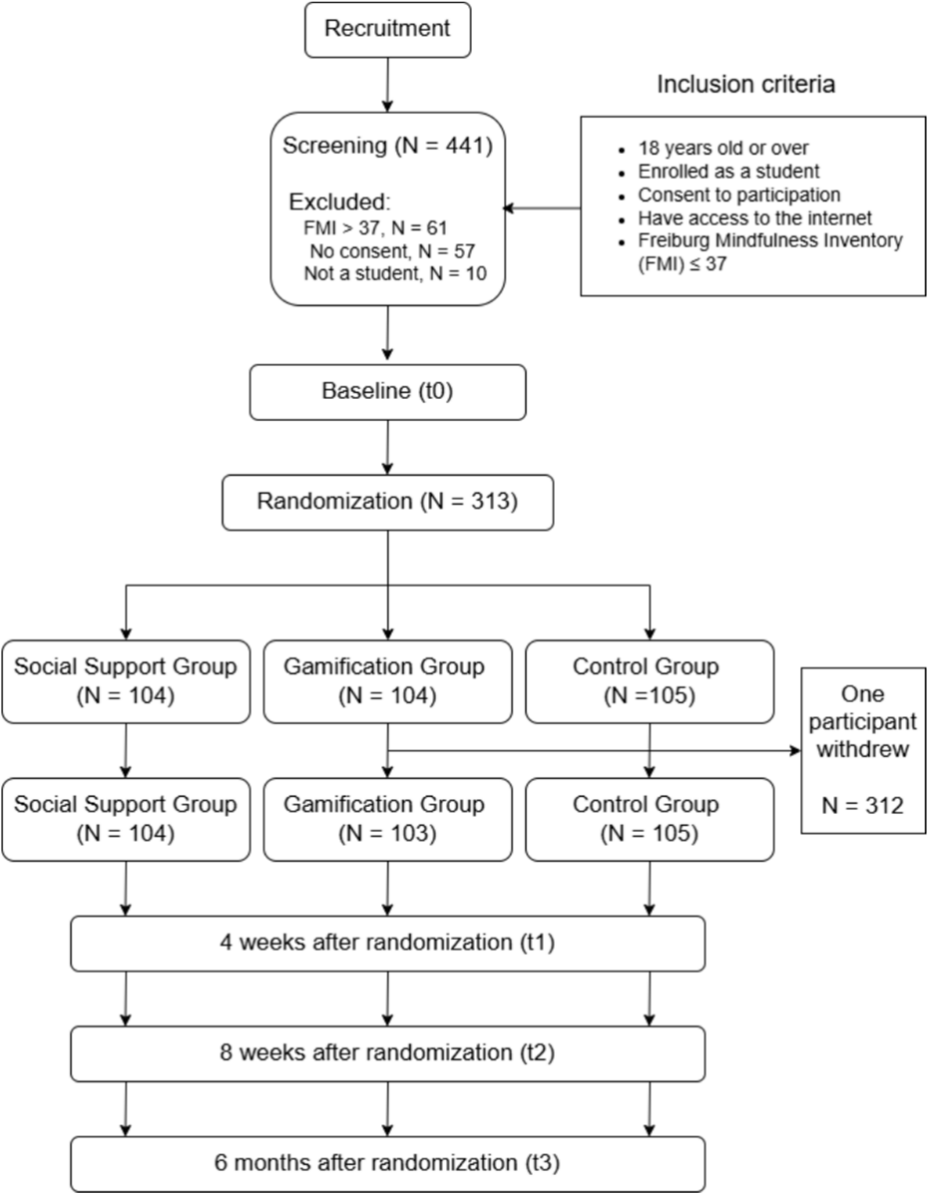
Study design

This study adopts a parallel, three-arm RCT design to assess the superiority of two intervention groups, namely the Gamification Group (GG) and Social Support Group (SSG), compared to the Control Group (CG). The trial includes one screening and three additional time points (see Fig. 1). Additionally, exploratory comparisons among the three intervention versions were conducted to investigate potential differences in user engagement and usability. All participants in the three groups received the same StudiCare mindfulness intervention. However, the interventions in GG and SSG were enhanced with PD optimization. The RCT was conducted within the StudiCare project, which was funded by BARMER health insurance. StudiCare offers IMIs that address psychological and behavioral issues among university students, including resilience, physical activity, stress, procrastination, and anxiety. The project is part of the WHO World Mental Health International College Student (WMH-ICS) initiative.^1^ Clinical outcomes were also evaluated as part of the trial, but these results will be reported elsewhere.

### Eligibility Criteria

To participate in the study, individuals had to be at least 18 years old, enrolled in a university, demonstrate sufficient knowledge of German or English, give their consent to participate through a digital informed consent opt-in procedure integrated into the online recruitment and baseline survey via Unipark (https://www.unipark.com), have access to the internet, own a smartphone, and have a medium to low level of mindfulness, as determined by a score of $\le 37$ on the short questionnaire of the Freiburg Mindfulness Inventory (FMI) [16]. No specific exclusion criteria were applied.

### Setting and Enrollment

Participants were recruited through various channels. At Ulm University, recruitment efforts were carried out by sending e-mails to the test subjects’ mailing list and placing flyers and posters throughout the campus. Additionally, recruitment initiatives were extended to several universities in Germany as part of the StudiCare project. Through collaboration with these institutions, students were regularly informed via email about ongoing interventions. All recruitment activities directed potential participants to the project’s website, where comprehensive information and contact details for the study team were provided. Furthermore, recruitment efforts were expanded through:

- Utilization of social networks, including the StudiCare Instagram page.
- Engagement with other universities in Germany not directly involved in the StudiCare project, including interactions with student councils and mailing lists.
- Collaboration with psychosocial counseling centers at various colleges and universities in Germany.

Upon submission of a consent form, participants underwent a baseline assessment before proceeding to randomization.

### Intervention

The StudiCare Mindfulness intervention was delivered via the eSano eHealth platform, a research-based system designed for IMIs. The platform comprises three core components: (1) a Content Management System for authoring and updating intervention materials; (2) an eCoach interface that handles study administration and guides participants through the intervention; and (3) a cross-platform participant app that lets users access their course content on any device. Further details are available in [17, 18].

StudiCare Mindfulness is an improved version of the original intervention described in [19]. The intervention consists of six core modules, two additional modules, and two refresher modules. The core modules are made available one after the other, i.e., each module can only be started after the previous module has been completed. Participants are advised to complete each module weekly and to spend about 45 to 60 min on each module. However, participants were also free to complete the intervention at their own pace. After completing the six core modules, participants were able to access the two additional modules. In addition, the first refresher module became available 4 weeks after completing the sixth core module. The second refresher module was unlocked 8 weeks later. The first module introduces the structure and goals of the intervention. The second module focuses on cultivating awareness of the present moment, while the third module is concerned with how to detach from stress-inducing thoughts. The fourth module strengthens positive thinking patterns through structured exercises. The fifth module guides participants to identify their personal core values and align their actions with those values. The sixth module focuses on self-care and self-compassion, using mindfulness techniques to promote self-acceptance and emotional regulation. The first additional module explores the connection between body and mind and encourages participants to improve their body awareness. The second additional module develops mindful awareness of bodily signals such as heart rate and hunger in order to better recognize stress reactions. The first refresher module reviews the content of modules 2 to 4 and supports ongoing mindfulness practice. The second refresher module picks up on modules 5 to 6 and helps participants to evaluate their progress and overcome obstacles to maintaining mindfulness.

### PD Conditions

In the gamification group, PD optimisation was implemented through a skill system and gamified infographics that were integrated into various intervention components. Research indicates that superficial reward systems can undermine intrinsic motivation; therefore, we chose an approach centered on competence-building elements to strengthen participants’ intrinsic motivation [20]. The infographics components are located on the home page, in the intervention overview, in the lesson overview, and on the lesson pages. They visually indicate the completion of a lesson and provide participants with an overview of their progress during the intervention. In addition, the progress bar on the lesson page allows the intervention creators to add custom text, such as encouraging phrases, to promote student engagement. The progress page has three main components: Intervention Progress, which displays the capability polar chart of all interventions with capabilities; Lesson Progress, which lists the in-progress lessons with progress bars enabled sorted by highest lesson progress; and Capability Progress, which lists the unfinished capabilities sorted by highest capability progress. Each skill includes attributes such as title, color for identification, optional icon to represent the skill, and an optional description to clarify various aspects. The attributes and progress of the skills are displayed on the skills page, which is accessed after completing the lesson. The overall progress of the skill is defined as the ratio of completed lessons to all available lessons within the skill.

In the social support group, the PD optimization is based on pairing participants as supportive buddies within the same group, establishing a collaborative partnership from the outset. This implementation was based on social accountability, a principle shown to promote goal attainment [21]. Furthermore, the implementation used automated, pre-set emails to create a controlled and safe environment, a necessary precaution given the risks of unmoderated communication in eHealth settings [22]. Each participant’s designated buddy is intended to act as a supportive companion throughout the intervention. Once teamed up, participants receive an initial introduction email announcing that a partner has been found and encouraging the use of the collaborative support feature. Both individuals are expected to complete a daily diary entry, with the overarching goal of reinforcing consistent engagement with the intervention. As participants progress, the system monitors when each partner completes their daily entry. If both partners complete their entries, both receive a congratulatory email acknowledging their mutual effort. If only one partner finishes, that participant gains the option to send a single daily reminder email to encourage the other partner to complete the task. Although participants can record multiple entries in a single day, the emphasis is on ensuring at least one meaningful completion per day. These supportive reminders and congratulatory messages are exchanged exclusively via email and can only be triggered once per day per individual. They are pre-configured and unalterable, maintaining consistency, reliability, privacy, and preventing the exchange of inappropriate content.

In both groups (i.e., GG and SSG) participants were introduced to these PD features from the first module. Therefore, they had the chance to utilize gamification or social support strategies from the very beginning of the intervention. Participants in the CG received only the standard StudiCare Mindfulness intervention, without any gamification or social support strategies.

### Assessments and Outcomes

Participant assessments were conducted at multiple time points: baseline ($t_0$), 4 weeks ($t_1$; intermediate), 8 weeks ($t_2$; post-intervention), and 6 months ($t_3$; follow-up). Various instruments gathered data on socio-demographic characteristics, intervention adherence (primary outcome), engagement, and usability (secondary outcomes). At baseline ($t_0$), participants completed a socio-demographic questionnaire, providing information on age, gender, marital status, study semester, and study program.

#### Primary Outcome: Adherence

Adherence to the intervention, the primary outcome, was measured at the 8-week mark ($t_2$) by tracking participants’ completion of intervention modules. Participants who completed at least five out of six core lessons were considered adherent, aligning with similar studies on web-based mindfulness interventions. For example, the review by Winter et al. [23] considered 80% program completion as an adherence standard, which corresponds to completing approximately five out of six core modules in this study. No changes to the pre-specified primary outcome were made after the trial commenced.

#### Secondary Outcomes

Engagement was assessed at $t_2$ using platform data, including:

- Total time spent on the intervention.
- Total modules completed: a continuous count (0-10) of all module types (core, additional, and refresher) completed by participants over the full study period.
- Number of completed daily tasks.
- Total time spent on diary entries.
- Number of logins.

The measures for integration were compared across groups in the first 8 weeks after randomization. Additionally, engagement was further assessed by tracking:

- Number of completed additional modules (maximum of two modules, available after $t_2$).
- Number of completed refresher modules (maximum of two modules, available at $t_3$).

Moreover, the relationship between the number of diary entries and module completion was analyzed to explore whether increased diary use correlated with higher module completion. Additionally, usability and other user experience aspects were evaluated at $t_2$ using the System Usability Scale (SUS) [24] and a self-constructed questionnaire. The self-constructed questionnaire, composed of 18 items, was used to assess various dimensions of user experience. These items were organized into four categories: usability (4 items); user engagement (5 items); evidence-based content (5 items); and visual design (4 items). Responses to these items were rated on a 5-point Likert scale ranging from 1 (“strongly disagree”) to 5 (“strongly agree”). Further assessments were conducted at $t_1$ and $t_3$ to evaluate intervention effectiveness using tools such as the Freiburg Mindfulness Inventory (FMI), Patient Health Questionnaire (PHQ-8), Generalized Anxiety Disorder Questionnaire (GAD-7), and Well-being Questionnaire (WHO-5). However, data from $t_1$ and $t_3$ are excluded from this paper and will be analyzed in a separate publication focused on intervention effectiveness.

### Sample Size

The required sample size was calculated using G*Power [25] to detect an expected increase in adherence. This was measured as the number of completed modules in the intervention groups (SSG and GG) compared to CG at time point $t_2$. Based on the meta-analysis by Baumeister et al. [26], a moderate effect size was reported for guided compared to unguided Internet-based interventions ($\text {SMD} = 0.52, 95\% \, \textit{CI}\ [0.37, 0.67]$). Considering that PD elements, such as the social support and gamification components, represent a technological, low-dose form of guidance, an effect size of $d = 0.40$ was assumed. A power analysis (two-tailed) indicated that a sample size of $N = 133$ participants per group (total $N = 387$) would provide 90% power to detect this effect with an alpha level of $\alpha = 0.05$.

### Randomization

After the baseline assessment, randomization of participants was carried out by a researcher who was otherwise not involved in the study in order to avoid selection bias and concealment bias. Randomization was carried out according to an allocation ratio of 1:1:1 and used block randomization, with varying block sizes (6, 9, and 12) being used. The randomization list was generated using the computer-based program Sealed Envelope (https://www.sealedenvelope.com/). Participants were partially blinded; they were not informed of the specific study arm assignments. In the SSG, participants were notified of the available social support feature and encouraged to use it, while GG participants could directly observe gamification elements. However, the comparative focus on these features was not disclosed.

### Ethical Approval

This trial received ethical approval from the Ethics Committee at Ulm University, Ulm, Germany (296-22, date: 07.09.2022), and was preregistered with the German Clinical Trials Register (DRKS00030975). It adhered to the CONSORT Guidelines for Noninferiority and Equivalence Trials Studies [27]. All participants provided detailed informed consent, confirming their understanding of the study procedures and their rights, including the freedom to withdraw at any point without consequences. This work does not include any personally identifiable information or images.

### Statistical Methods

This analysis was conducted using an intention-to-treat (ITT) approach, with participants analyzed in their originally assigned groups. For the primary outcome of adherence, a Generalized Linear Model (GLM) with a Negative Binomial distribution was selected to address anticipated overdispersion. CG served as the reference category, with statistical comparisons made between CG and GG, and between CG and SSG. Statistical significance was determined using *p*-value, and 95% confidence intervals were calculated for the group comparisons. For secondary outcomes related to engagement metrics, GLM with a Zero-Inflated Negative Binomial distribution was used, given the anticipated overdispersion and presence of zero values. Group comparisons were conducted (CG vs GG, CG vs SSG, GG vs SSG), with CG as the reference category. Statistical significance was evaluated using *p*-value, with 95% confidence intervals calculated for each group comparison. To investigate the relationship between the number of diary entries and the number of modules completed, Pearson’s correlation coefficient was calculated. Additionally, models such as Polynomial Regression, Decision Tree, Random Forest, K-Nearest Neighbors (KNN), and Elastic Net were used to explore potential associations. Model performance was assessed using Mean Squared Error (MSE) and the coefficient of determination ($R^2$) following an 80/20 train-test split, supplemented by 5-fold cross-validation. Usability data from the SUS were analyzed using the Kruskal-Wallis test to assess overall group differences. For pairwise group comparisons, the Mann-Whitney U test was conducted due to non-parametric data characteristics. Additionally, for the self-constructed questionnaire, a composite score for each category was calculated as the mean of relevant item responses. Analyses for questionnaire-based outcomes were performed on available data only, as no imputation was applied for missing responses.

## Results

### Participants

A total of 313 participants initially qualified for the study based on a baseline FMI score of $\le 37$. Following randomization, one participant withdrew and requested complete data removal, resulting in a final sample of 312 participants. These participants were distributed randomly among the three intervention groups: 103 in GG, 104 in SSG, and 105 in CG. The average age across all participants was ($M = 26.3$, *SD* = 5.9), with the CG group being slightly older on average ($M = 27.4$, *SD* = 7.3) compared to the GG ($M = 26.2$, *SD* = 5.6) and SSG ($M = 25.4$, *SD* = 4.4). The majority (78.8%) of participants were female (*N* = 246), with a balanced distribution across the groups (GG: 76 (73.7%), SSG: 82 (78.8%), CG: 88 (83.8%)). Most participants (88.7%) were full-time students (*N* = 277) and the average number of completed semesters was ($M = 10.3$, *SD* = 6.3). While the groups had comparable distributions in terms of study program and citizenship, the SSG group had a noticeably higher proportion of students in education. The majority (82.6%) of participants were German nationals (*N* = 258). For further sociodemographic details, see Table 1.

**Table 1:** Baseline characteristics

	Total	GG (*N* = 103)	SSG (*N* = 104)	CG (*N* = 105)
Age (M (SD))	26.3 (5.9)	26.2 (5.6)	25.4 (4.4)	27.4 (7.3)
Female	246 (78.8%)	76 (73.7%)	82 (78.8%)	88 (83.8%)
Single	209 (66.9%)	69 (66.9%)	71 (68.2%)	69 (65.7%)
Not in therapy	162 (51.9%)	59 (57.2%)	51 (49%)	52 (49.5%)
Study				
Full time student	277 (88.7%)	91 (88.3%)	92 (88.4%)	94 (89.5%)
No. of semesters (M (SD))	10.3 (6.3)	10.4 (6.8)	10.3 (5.6)	10.1 (6.3)
study Program				
Social sciences	60 (19.2%)	24 (23.3%)	17 (16.3%)	19 (18%)
Education	57 (18.2%)	15 (14.5%)	24 (23%)	18 (17.1%)
Natural sciences	42 (13.4%)	10 (9.7%)	20 (19.2%)	12 (11.4%)
Medicine	30 (9.6%)	10 (9.7%)	8 (7.6%)	12 (11.4%)
Psychology	25 (8%)	10 (9.7%)	7 (6.7%)	8 (7.6%)
Citizenship				
German	258 (82.6%)	84 (81.5%)	88 (84.6%)	86 (81.9%)
Swiss	8 (2.5%)	2 (1.9%)	1 (0.9%)	5 (4.7%)
Chinese	5 (1.6%)	2 (1.9%)	-	3 (2.8%)
Mexican	3 (0.9%)	3 (2.9%)	-	-
Italian	3 (0.9%)	-	1 (0.9%)	2 (1.9%)

### Recruitment

Recruitment took place from December 2022 to February 2024. Despite not reaching the target sample size, recruitment was concluded after exhausting all available resources allocated for recruitment. Participants were assessed at baseline ($t_0$) and at 8 weeks post-intervention ($t_2$) for the primary outcomes presented here.

### Primary Outcome

No significant differences in adherence were observed between the control group and the two intervention groups (GG or SSG). The mean number of modules completed was ($M = 2.52$, *SD* = 1.92) for CG, ($M = 2.69$, *SD* = 1.89) for GG, and ($M = 2.76$, *SD* = 2.03) for SSG. Specifically, the number of modules completed neither differ significantly between CG and GG ($\beta = 0.06$, 95% *CI* $[-0.10, 0.23]$, $p = 0.45$), nor between CG and SSG ($\beta = 0.09$, 95% *CI* $[-0.07, 0.25]$, $p = 0.28$).

**Fig. 2:**
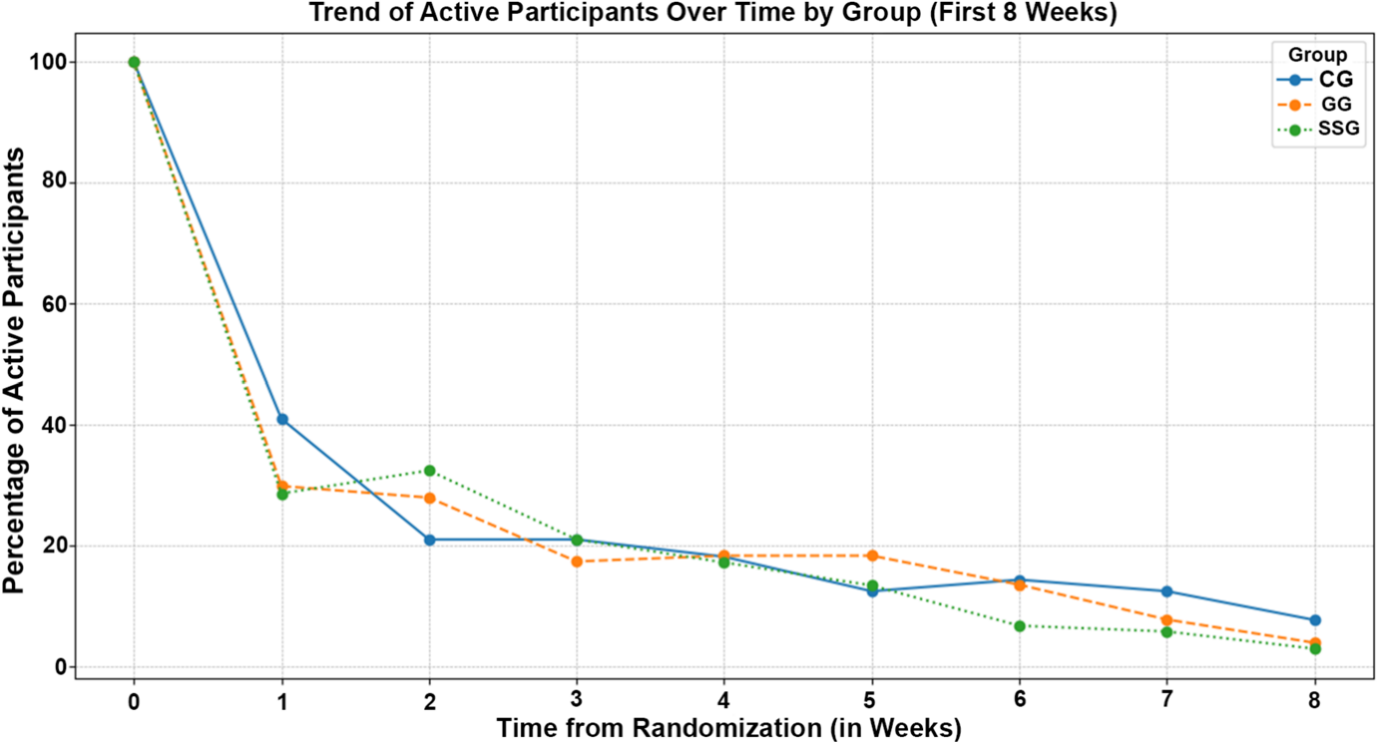
Number of active participants

Additionally, Fig. 2 shows the percentage of active participants (completing at least a module in a given week) within the first 8 weeks after randomization for each group (CG, GG, and SSG). At the start of the intervention, all groups started with a comparable number of participants: CG with 105, GG with 103, and SSG with 104 participants. However, there was a rapid decline in the percentage of active participants during the first week in all groups. By the end of the first week, the percentage of active participants dropped significantly to 40.95% in CG, 29.81% in GG, and 28.57% in SSG. This declining trend continued over the subsequent weeks, with the percentage of active participants stabilizing somewhat after the fourth week. By the eighth week, the percentage of active participants fell to 7.62% in CG, 3.85% in GG, and 2.86% in SSG.

### Secondary Outcome

No significant differences were observed in the total time spent on the intervention or the total modules completed over the full study period between CG and GG ($\beta = -0.08$, 95% *CI* $[-0.35, 0.18]$, $p = 0.54$). Similarly, no significant differences were noted between CG and SSG in these metrics ($\beta = -0.05$, 95% *CI* $[-0.32, 0.21]$, $p = 0.67$). However, SSG participants spent significantly more time on diary entries compared to CG ($\beta = 0.42$, 95% *CI* [0.12, 0.72], $p = 0.001$) and completed more diary entries ($\beta = 0.54$, 95% *CI* [0.21, 0.87], $p < 0.001$). Furthermore, no differences were found in the number of additional modules or refresher modules completed across the groups. Table 2 presents the descriptive statistics (Mean and Standard Deviation) for the engagement metrics in the study groups, while Table 3 provides the results of the group comparisons, including coefficients, confidence intervals, and *p*-values.

**Table 2:** Descriptive statistics for engagement measures of study groups

Engagement measure	CG (M ± SD)	GG (M ± SD)	SSG (M ± SD)
total_modules_completed	3.14 ± 2.50	3.06 ± 2.46	3.18 ± 2.62
modules_minutes	189.73 ± 194.43	174.26 ± 181.24	178.93 ± 190.13
diary_minutes	3.44 ± 9.64	3.35 ± 8.95	5.26 ± 13.11
number_diaries	1.63 ± 4.24	1.68 ± 4.42	2.82 ± 6.33
number_diaries_in_first_8_weeks	1.44 ± 3.93	1.57 ± 4.32	2.20 ± 4.93
number_logins	4.94 ± 4.91	5.34 ± 4.95	5.58 ± 7.19
number_logins_in_first_8_weeks	3.47 ± 3.21	4.07 ± 4.82	3.96 ± 5.60
number_additional_modules	0.12 ± 0.43	0.15 ± 0.50	0.21 ± 0.58
number_refresher_modules	0.10 ± 0.35	0.13 ± 0.42	0.12 ± 0.43

**Table 3:** Engagement comparison of study groups

Measure	CG vs GG			CG vs SSG			GG vs SSG		
	($\beta$)	95% CI	$p$	($\beta$)	95% CI	$p$	($\beta$)	95% CI	$p$
total_modules_completed	$-$0.02	[$-$0.33; 0.28]	0.86	0.01	[$-$0.29; 0.32]	0.94	0.03	[$-$0.40; 0.47]	0.86
modules_minutes	$-$0.08	[$-$0.35; 0.18]	0.54	$-$0.05	[$-$0.32; 0.21]	0.67	0.02	[$-$0.35; 0.41]	0.89
diary_minutes	$-$0.02	[$-$0.33; 0.28]	0.87	0.42	[0.12; 0.72]	0.01	0.45	[0.02; 0.88]	0.04
number_diaries	0.03	[$-$0.31; 0.37]	0.85	0.54	[0.21; 0.87]	0.00	0.51	[0.04; 0.99]	0.03
number_diaries_in_first_8_weeks	0.08	[$-$0.26; 0.43]	0.63	0.42	[0.08; 0.76]	0.01	0.33	[$-$0.14; 0.82]	0.17
number_logins	0.07	[$-$0.21; 0.37]	0.61	0.12	[$-$0.17; 0.41]	0.42	0.04	[$-$0.37; 0.46]	0.83
number_logins_in_first_8_weeks	0.15	[$-$0.14; 0.46]	0.31	0.13	[$-$0.17; 0.43]	0.39	$-$0.02	[$-$0.45; 0.40]	0.91
number_additional_modules	0.21	[$-$0.56; 0.99]	0.59	0.52	[$-$0.21; 1.26]	0.16	0.30	[$-$0.76; 1.38]	0.57
number_refresher_modules	0.34	[$-$0.50; 1.20]	0.43	0.26	[$-$0.60; 1.13]	0.55	$-$0.08	[$-$1.30; 1.13]	0.89

### Effect Size

Table 4 shows Cohen’s d ($\delta$) and 95% confidence intervals (CI) for the primary outcome and secondary outcomes in the study groups (CG vs GG, CG vs SSG, GG vs SSG).

**Table 4:** Cohen’s d values ($\delta$) and 95% confidence intervals (CI) for the primary and secondary outcomes

Measure	CG vs GG		CG vs SSG		GG vs SSG	
	$\delta$	95% CI	$\delta$	95% CI	$\delta$	95% CI
number_modules_in_first_8_weeks	0.08	[$-$0.18; 0.35]	0.12	[$-$0.15; 0.39]	0.03	[$-$0.23; 0.30]
total_modules_completed	$-$0.03	[$-$0.30; 0.23]	0.01	[$-$0.25; 0.28]	0.48	[$-$0.22; 0.31]
modules_minutes	$-$0.08	[$-$0.35; 0.18]	$-$0.05	[$-$0.32; 0.21]	0.02	[$-$0.24; 0.29]
diary_minutes	$-$0.01	[$-$0.28; 0.26]	0.15	[$-$0.11; 0.42]	0.17	[$-$0.10; 0.44]
number_diaries	0.01	[$-$0.25; 0.28]	0.22	[$-$0.05; 0.49]	0.20	[$-$0.06; 0.47]
number_diaries_in_first_8_weeks	0.03	[$-$0.23; 0.30]	0.17	[$-$0.10; 0.44]	0.13	[$-$0.13; 0.40]
number_logins	0.07	[$-$0.19, 0.35]	0.10	[$-$0.16; 0.37]	0.03	[$-$0.23; 0.31]
number_logins_in_first_8_weeks	0.14	[$-$0.12; 0.41]	0.10	[$-$0.16; 0.37]	$-$0.02	[$-$0.29; 0.25]
number_additional_modules	0.06	[$-$0.20; 0.33]	0.16	[$-$0.10; 0.43]	0.10	[$-$0.16; 0.37]
number_refresher_modules	0.10	[$-$0.16; 0.37]	0.07	[$-$0.19; 0.34]	$-$0.02	[$-$0.29; 0.24]

### Adverse Events

No adverse events or unintended effects were reported in any of the study groups.

### Additional Analysis

**Fig. 3:**
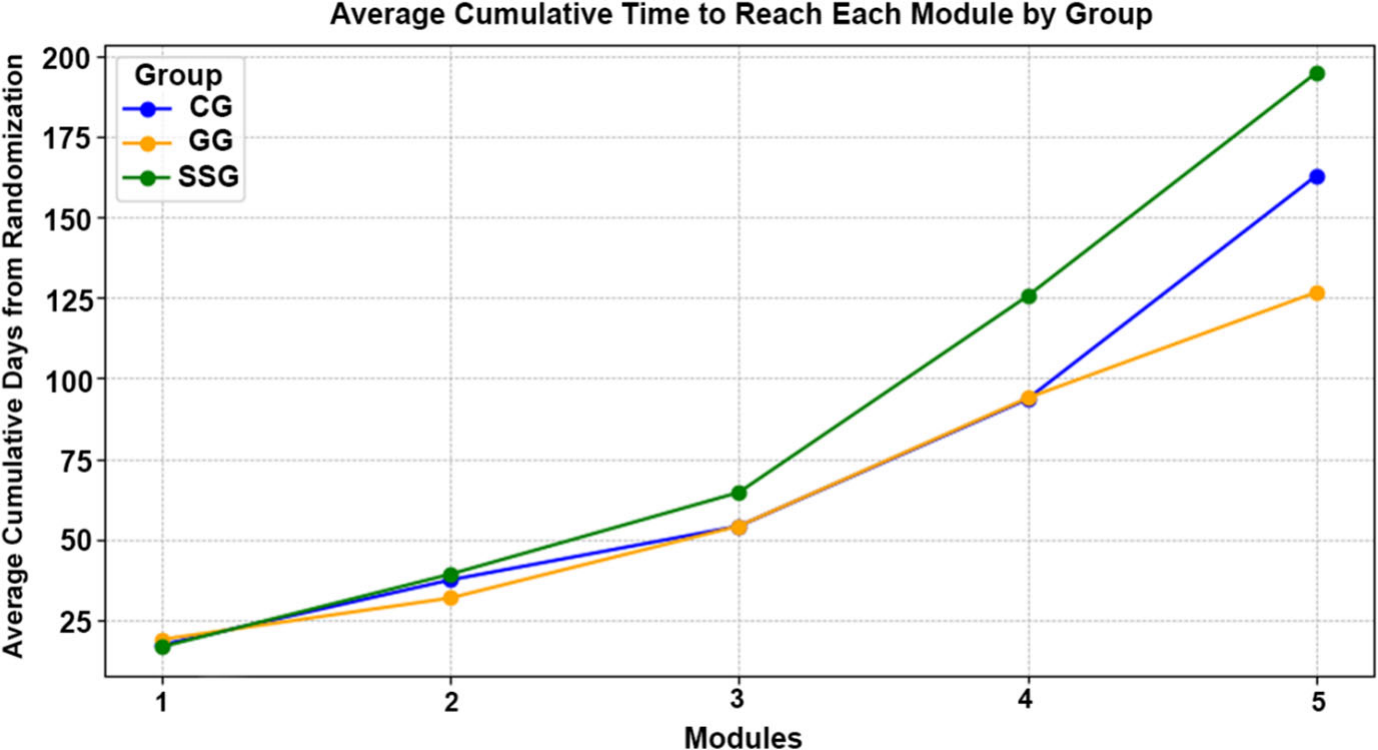
Average cumulative days to reach each module by group

Figure 3 shows the average cumulative completion time for participants to finish each module in the three groups. Initially, all groups started with similar times to complete the first module: CG completed it in 17.11 days, GG in 18.81 days, and SSG in 16.52 days. As the participants progressed through the intervention, the average cumulative days required to complete each subsequent module increased. By the fifth module, CG required an average of 162.93 cumulative days, GG needed 126.75 days, and SSG took the longest at 194.96 days.

The relationship between the number of diary entries and completed modules was examined in the full sample as well as individual study groups. In the full sample, the correlation was $r = 0.42$ ($p = 5.86 \times 10^{-15}$). For GG, the correlation was $r = 0.40$ ($p = 2.41 \times 10^{-5}$), while for CG, it was $r = 0.35$ ($p = 0.00017$). In SSG, the correlation was highest at $r = 0.49$ ($p = 1.10 \times 10^{-7}$). Model performance varied in the full sample and individual groups. In the full sample, KNN performed best with an MSE of 4.00 and $R^2$ of 0.35, while Polynomial Regression (degree 3) provided the best fit in Group SSG, with an MSE of 2.74 and $R^2$ of 0.29. Performance was generally poorer in GG, with Linear Regression and KNN yielding negative $R^2$ values. Furthermore, for Linear Regression, the slope for the full sample was 0.20 (Intercept: 2.65, $p < 0.0001$). In GG, the slope was 0.19 (Intercept: 2.43, $p = 0.0017$), for SSG, the slope was 0.20 (Intercept: 2.73, $p < 0.0001$), while for CG, the slope was 0.20 (Intercept: 2.71, $p = 0.0001$). These slope values represent the rate of change in the total modules completed for each additional diary entry. Table 5 provides detailed results for each model, including MSE and $R^2$ values for both train-test split (TT) and 5-fold cross-validation (5-F_CV).

**Table 5:** Correlation between diary entries and module completion

Model	Metric	Full Sample		GG		SSG		CG	
		TT	5-F_CV	TT	5-F_CV	TT	5-F_CV	TT	5-F_CV
Linear regression	MSE	5.51	5.26	7.34	5.70	3.38	6.43	8.21	5.72
	$R^2$	0.10	0.16	$-$0.04	0.05	0.13	$-$0.05	0.02	$-0.00^{*}$
Polynomial (Deg 2)	MSE	5.26	8.27	7.54	4.95	4.31	71.19	7.88	40.22
	$R^2$	0.14	$-$0.19	$-$0.07	0.14	$-$0.10	$-$5.94	0.06	$-$7.38
Polynomial (Deg 3)	MSE	5.01	26.06	7.66	5.52	2.74	4.98	7.60	708.12
	$R^2$	0.18	$-$2.36	$-$0.09	0.04	0.29	0.12	0.09	$-$150.83
Decision tree	MSE	4.45	5.05	9.28	6.54	4.20	6.25	6.13	4.14
	$R^2$	0.27	0.19	$-$0.32	$-$0.16	$-$0.07	$-$0.17	0.27	0.25
Random forest	MSE	4.47	4.99	8.32	6.02	3.81	5.52	5.73	4.13
	$R^2$	0.27	0.20	$-$0.19	$-$0.03	0.02	$-$0.04	0.31	0.25
KNN	MSE	4.00	5.18	8.67	6.01	4.70	5.29	6.31	5.04
	$R^2$	0.35	0.17	$-$0.24	$-0.00^{*}$	$-$0.19	0.04	0.25	0.12
Elastic Net	MSE	5.55	5.25	7.61	5.66	3.39	5.91	8.23	5.59
	$R^2$	0.10	0.16	$-$0.08	0.05	0.13	$-$0.01	0.02	0.02

*Note.* Values marked with * indicate entries where the absolute value is less than 0.005

An exploratory analysis was conducted to investigate the distribution and differences in SUS scores in the three study groups: SSG ($N = 34$), GG ($N = 34$), and CG ($N = 35$). This analysis utilized a Kruskal-Wallis test followed by pairwise Mann-Whitney U tests. The Kruskal-Wallis test revealed no statistically significant differences in SUS scores between groups ($H = 1.708$, $p = 0.426$). This indicates that the median SBS values were comparable in all groups. Further exploratory analysis with pairwise comparisons also indicated no statistically significant differences:

- SSG vs. CG: $U = 720.500$, $p = 0.265$
- GG vs. CG: $U = 719.000$, $p = 0.273$
- SSG vs. GG: $U = 513.000$, $p = 0.995$

These findings indicate no significant differences in system usability perceptions among participants in different groups. The self-constructed questionnaire results indicate that the SSG ($N = 34$) group reported the highest mean scores for usability (*M* = 4.40) and visual design (*M* = 3.88). Conversely, the GG ($N = 34$) group exhibited the highest mean score in the evidence-based category (*M* = 4.44). In contrast, the CG ($N = 35$) group consistently had the lowest averages in most of the measured categories, as listed in Table 6.

**Table 6:** Results for self-constructed questionnaire

Category	Overall			GG			SSG			CG		
	M ± SD	Min	Max	M ± SD	Min	Max	M ± SD	Min	Max	M ± SD	Min	Max
Usability	4.31 ± 0.68	2.25	5.00	4.22 ± 0.68	2.50	5.00	4.40 ± 0.70	2.25	5.00	4.31 ± 0.68	2.50	5.00
User engagement	3.36 ± 0.59	2.00	4.80	3.44 ± 0.66	2.20	4.80	3.50 ± 0.44	2.60	4.40	3.18 ± 0.59	2.00	4.80
Evidence-based	4.31 ± 0.54	2.60	5.00	4.44 ± 0.50	3.20	5.00	4.36 ± 0.53	3.40	5.00	4.15 ± 0.54	2.60	5.00
Visual design	3.78 ± 0.65	2.25	5.00	3.69 ± 0.70	2.25	5.00	3.88 ± 0.63	3.00	5.00	3.78 ± 0.62	2.50	5.00

## Discussion

### Primary Outcome

The results show no significant differences in adherence between the groups, with comparable average numbers of completed modules in CG, GG, and SSG. This suggests that the implemented gamification and social support strategies did not significantly impact adherence when compared to CG. Specifically, our observation that a gamification strategy combining a skill system and progress infographics failed to increase adherence aligns with the mixed results reported in systematic reviews of gamification in eHealth interventions. For example, the review of De Croon et al. [28] reports that 19 out of 27 studies found that gamification had a positive effect on adherence, representing relatively strong evidence of its potential impact. However, the definition of adherence remains inconsistent in the reviewed studies, which could account for the variability in outcomes in different contexts. A likely explanation for these null findings is the demanding nature of the core intervention. “StudiCare Mindfulness” is a comprehensive program with six core modules, each requiring a 45 to 60-min weekly commitment. The inherent workload and complexity of the IMI were likely the dominant factors influencing user adherence, potentially overpowering the more subtle effects of the added PD strategies. Therefore, the observed lack of effect may not indicate that these PD strategies are ineffective universally, but rather that their influence was not compelling enough to alter engagement with a high-demand intervention of this kind. These results are supported by other findings in the literature, such as those showing that while gamification can increase physical activity (PA), the results are often modest and influenced by heterogeneity in the design of interventions [29]. The rapid decline in active participants during the first week of the intervention may indicate a need for more intensive strategies early in the intervention to prevent disengagement. Also, the passive nature of some parts of eHealth interventions may contribute to the dropout rate if users do not feel actively involved in the intervention [30]. Future interventions might benefit from focusing on early-stage engagement, using more adaptive and interactive strategies to retain users from the outset. The findings suggest that while gamification and social support strategies implemented in this study may have some potential, their effects on adherence in this context were limited. Moreover, the incremental effect of adding a single PD strategy, such as those tested here, to an already effective intervention may be quite small (e.g., Cohen’s $d \sim 0.05$). Expecting moderate or large increments from a single component could be unrealistic [12]. Rather, multiple small incremental benefits from several PD strategies might accumulate over time [31]. Detecting these subtle improvements would likely require more sensitive study designs [12, 32]. More tailored and context-specific strategies, such as those targeting early-phase engagement and addressing the multiple dimensions of user experience, may be necessary to enhance adherence in digital health interventions.

### Secondary Outcomes

The analysis of the secondary outcomes revealed no significant differences in most engagement metrics, such as module completion and time spent in the study groups. This suggests that the implemented gamification and social support strategies did not strongly influence overall engagement. The significant increase in diary entries and time spent on diary entries in the SSG group points to a more nuanced effect of the automated email-based social support strategy. The social support strategy in SSG specifically aimed to promote diary completion through peer-driven reminders and feedback. Although the confidence intervals are relatively broad, indicating some uncertainty, the results suggest that this targeted approach did contribute to increased engagement with the diary feature. However, the increased engagement with the diaries did not extend to other metrics such as module completion or overall intervention adherence, demonstrating the challenge of translating targeted engagement into improved adherence. This outcome is consistent with multidimensional models that conceptualize engagement by distinct behavioral facets, such as usage “depth” with a specific feature versus overall program “amount” [33]. The weak predictive relationship between these different facets is also seen in other interventions where, for example, initial engagement scores failed to predict subsequent logins [34].

Interestingly, no differences were found in other engagement metrics, such as the number of logins, additional modules, or refresher modules completed. This suggests that the social support strategy enhanced engagement with specific behaviors (the diaries) but did not generate broader engagement. This indicates that there can be limitations in focusing on single engagement strategies. Therefore, a more holistic approach to the application of social support within eHealth interventions may be needed, so that it enhances engagement with multiple aspects of the intervention and supports overall adherence. A multilayered approach combining various engagement strategies, such as social support, gamification, and personalized feedback, may promote a more motivating user experience.

The analysis of the association between diary entries and module completion shows that while diary engagement positively correlates with module completion in all groups, this relationship did not translate into broader improvements in adherence. The strong correlation in SSG, where the social support strategy specifically targeted diaries, reinforces earlier findings that engagement with one aspect of the intervention may not be enough to drive comprehensive adherence. The varying performance of predictive models, especially the better fit of Polynomial Regression (degree 3) in SSG, indicates that the relationship between diaries and module completion may involve more complex, non-linear dynamics, particularly when influenced by this type of automated social support. This supports the thesis that interventions should explore multi-pronged strategies that address both specific behaviors and more holistic engagement throughout the intervention. While the implemented PD strategies, such as gamification and social support, can be useful tools, other factors—such as the content, difficulty, or length of the modules—are likely to play important roles in influencing adherence. Therefore, a combination of engagement strategies, paired with thoughtful content design, may be necessary to drive both adherence and overall engagement in future interventions.

The SUS results for SSG, GG, and CG suggest that the integration of the implemented social support and gamification strategies did not negatively affect the perceived usability of the platform. The absence of significant differences in pairwise comparisons further indicates that these features did not disrupt the basic user experience. However, subtle variations were observed in the self-constructed questionnaire, particularly in perceptions of usability, user engagement, evidence-based content, and visual design. SSG reported higher scores in usability and visual design, while GG scored highest in the evidence-based category, suggesting that the specific form of gamification tested may enhance the perception of credibility. In contrast, CG consistently scored lower, potentially reflecting a less favorable perception of the platform without these features. The different effects in the various questionnaire categories suggest that different strategies can be used to target specific user needs. For example, gamification might improve perceived credibility, while social support could focus on enhancing usability. Further research could explore the factors contributing to these perceptions by investigating the different perceptions of the PD strategies through qualitative methods, such as think-aloud interviews.

## Limitations

This study faced several challenges. First, the sample size fell short of the target. Despite exhausting all recruitment strategies and resources over 13 months, only 313 of the required 399 participants were recruited and qualified via screening (with only 312 included in the final analysis). This deficit impacted the statistical power of the study, necessitating a cautious interpretation of the results. Although the trial was designed and conducted as a confirmatory study, the reduced sample size may limit the strength of the conclusions drawn from the data. Given that the incremental effects of single PD strategies, such as the specific implementations tested here, may be very small, the reduced sample size further limited the ability to detect such subtle differences. Additionally, the timing of the SUS and self-constructed questionnaires introduced another limitation. By the second time point ($t_2$), when these questionnaires were administered, the participant pool had diminished further, with only 103 out of the initial 313 providing responses. This substantial dropout rate of about 67% might skew the results, as it is uncertain whether the views of the respondents are representative of the initial cohort. Another limitation to consider is the potential for selection bias. Given that the remaining participants might represent a subgroup with either more favorable views of the intervention or different characteristics from those who dropped out, the results might not fully capture the varied responses of the initial sample. A final limitation is the sample’s gender composition. The predominance of female participants, while a consequence of open recruitment, is consistent with established research demonstrating that men are generally less likely than women to seek help for mental health and wellness issues [35]. This imbalance limits the generalizability of the findings, as the observed null effects may not extend to male populations who could respond differently to the tested PD strategies.

## Conclusion

This trial evaluated the potential impact of the implemented gamification and social support strategies on user adherence and engagement within the StudiCare Mindfulness digital intervention delivered through the eSano eHealth platform. While the gamification strategy did not lead to a marked increase in participation compared to the control group, the implemented social support enhanced engagement with diary-related activities. However, this increase in diary engagement did not translate into improved module completion rates. The usability ratings did not show any significant differences between the groups, indicating that the additional features did not negatively affect the platform’s usability. Nevertheless, the study’s smaller-than-anticipated sample size and high dropout rate warrant a cautious interpretation of the findings. Future research may benefit from larger samples and refined study designs to more clearly discern any subtle benefits of various PD strategies.

## Data Availability

The datasets generated and analyzed during this study are not publicly available due to necessary data security measures. These measures restrict access to authorized personnel only to maintain the confidentiality and protection of the data. Access to specific portions of these datasets may be granted under strict conditions and is subject to a rigorous approval process. Researchers who wish to access the data should contact the corresponding author. Requests will be reviewed on a case-by-case basis.

## Footnotes

1 https://www.hcp.med.harvard.edu/wmh/college_student_survey.php

## Code Availability

Data analysis was performed using Python 3.7 with key libraries including Pandas, NumPy, and Statsmodels. The code is not publicly available, but the statistical models and procedures are described in the Methods section.
