# Correction: Persuasive Design in a Digital Mindfulness Intervention: A Randomized Trial of a Skill-Based Achievement System and Automated Peer Encouragement

**DOI:** 10.1007/s41666-025-00225-5

**Published:** 2025-11-19

**Authors:** Abdul Rahman Idrees, Robin Kraft, Ann-Marie Küchler, Leandra Bantleon, Harald Baumeister, Manfred Reichert, Fanny Kählke, David Daniel Ebert, Rüdiger Pryss

**Affiliations:** 1 Institute of Databases and Information Systems, Ulm University, Ulm, Germany; 2 Department of Clinical Psychology and Psychotherapy, Ulm University, Ulm, Germany; 3 Institute of Clinical Epidemiology and Biometry, University of Würzburg, Würzburg, Germany; 4 Institute of Medical Data Science, University Hospital Würzburg, Würzburg, Germany; 5 Faculty of Applied Health Sciences, Deggendorf Institute of Technology, Deggendorf, Germany; 6 Professorship of Psychology and Digital Mental Health Care, Department of Sports and Health Sciences, Technical University of Munich (TUM), Munich, Germany

**Correction: Journal of Healthcare Informatics Research**

10.1007/s41666-025-00216-6

In the original online version of this article, the data availability statement was incorrect. Following the is revised statement:

The datasets generated and analyzed during this study are not publicly available due to necessary data security measures. These measures restrict access to authorized personnel only to maintain the confidentiality and protection of the data. Access to specific portions of these datasets may be granted under strict conditions and is subject to a rigorous approval process. Researchers who wish to access the data should contact the corresponding author. Requests will be reviewed on a case-by-case basis.

The original article was corrected.

